# A Strong Humoral Immune Response Induced by a Vaccine Formulation Containing rSm29 Adsorbed to Alum Is Associated With Protection Against *Schistosoma mansoni* Reinfection in Mice

**DOI:** 10.3389/fimmu.2018.02488

**Published:** 2018-11-02

**Authors:** Clarice Carvalho Alves, Neusa Araujo, Wilma Patrícia de Oliveira Santos Bernardes, Mariana Moreira Mendes, Sergio Costa Oliveira, Cristina Toscano Fonseca

**Affiliations:** ^1^Laboratório de Biologia e Imunologia de Doenças Infeciosas e Parasitárias, Instituto René Rachou, Fundação Oswaldo Cruz, Belo Horizonte, Brazil; ^2^Laboratório de Esquistossomose, Instituto René Rachou, Fundação Oswaldo Cruz, Belo Horizonte, Brazil; ^3^Laboratório de Imunologia de doenças Infeciosas, Instituto de Ciências Biológicas, Universidade Federal de Minas Gerais, Belo Horizonte, Brazil; ^4^Instituto Nacional de Ciências e Tecnologia em Doenças Tropicais, CNPq, MCT, Salvador, Brazil

**Keywords:** Sm29, alum, MPLA-SM, *Schistosoma mansoni*, vaccine, reinfection

## Abstract

The helminth *Schistosoma mansoni* is one of main causes of human schistosomiasis, a health and economic concern in some of the world's poorest countries. Current treatment regimens can lead to serious side effects and are not suitable for breastfeeding mothers. As such, efforts have been undertaken to develop a vaccine to prevent infection. Of these, Sm29 is a promising candidate that has been associated with resistance to infection/reinfection in humans and mice. Its ability to induce resistance to reinfection has also been recently demonstrated using a vaccine formulation containing Freund's adjuvant. However, Freund's adjuvant is unsuitable for use in human vaccines. We therefore evaluated the ability of Sm29 to induce protection against *S. mansoni* reinfection when formulated with either alum or MPLA as an adjuvant, both approved for human use. Our data demonstrate that, in contrast to Sm29 with MPLA, Sm29 with alum reduced parasite burden after reinfection compared to a control. We next investigated whether the immune response was involved in creating the differences between the protective (Sm29Alum) and non-protective (Sm29MPLA) vaccine formulations. We observed that both formulations induced a similar mixed-profile immune response, however, the Sm29 with alum formulation raised the levels of antibodies against Sm29. This suggests that there is an association between a reduction in worm burden and parasite-specific antibodies. In summary, our data show that Sm29 with an alum adjuvant can successfully protect against *S. mansoni* reinfection in mice, indicating a potentially effective vaccine formulation that could be applied in humans.

## Introduction

Schistosomiasis is one of the most important global helminthic infection, in terms of disability-adjusted life years (DALYs) (1, 2). Due to large effects on public health and productivity, a considerable amount of effort has been applied to finding candidates that could serve as an anti-schistosomiasis vaccine (3). One such promising vaccine candidate is the *Schistosoma mansoni* tegument protein Sm29. In areas endemic for schistosomiasis, high levels of circulating anti-Sm29 IgG1 and IgG3 have been associated with resistance to infection (4). Sm29 has also been tested using several experimental immunization protocols, consistently showing an ability to reduce worm burden. The first study that evaluated Sm29 with an experimental immunization protocol was published in 2008 and demonstrated a significant reduction in the number of adult worms after challenge (5). There was also an accompanying immune response that was defined by the production of high levels of IFN-γ and IgG1. Later, the protein was tested in combination with SmTSP-2 as a recombinant chimeric protein in order to more effectively potentiate the isolated antigens (6). Immunization with this chimeric protein also resulted in a significant reduction in parasite burden. In this case however, protection associated with high levels of specific IgG1 and IgG2a antibodies and a Th1 polarized immune profile, with significant production of IFN-γ and TNF-α. Finally, another chimeric protein consisting of Sm29 and Sm14 was also tested, showing protection against *S. mansoni* that was accompanied by significant production of IgG1 antibodies (7).

To further evaluate Sm29 as a vaccine candidate, we have recently tested this antigen in mice that were previously infected with *S. mansoni* and treated with praziquantel (8). Such pre-sensitization more accurately mimics the situation found in endemic areas in which the population is constantly being reinfected with the parasite. This study demonstrated that Sm29 with Freund's adjuvant provided protection against reinfection in these pre-sensitized animals (26–48%). Vaccination also induced an increase in overall antibody levels and a mixed cellular immune response (8). However, Freund's adjuvant is not recommended for use in humans due to high toxicity. Alternatively, alum adjuvants are widely used in human vaccine formulations (9), although only recently its mechanism of action begun to be elucidated. In 2008, two groups demonstrated that alum activates inflammasomes through a NLRP3 dependent-pathway (10, 11). Alum induces tissue damage that leads to uric acid release, enhancing the uptake of antigens by antigen presenting cells (APCs) (12). However, a more recent study has suggested that the effects of alum on the adaptive response may not involve NLRP3, but rather IL-2 production by dendritic cells (DCs). In the study, IL-2 released by DCs was dependent on phagocytosis, Syk, and NFAT activation. In the absence of IL-2-producing DCs, alum inoculation resulted in decreased proliferation of CD4^+^ T cells and decreased antibody production (13). Nevertheless, alum induces a Th2 type of immune response, irrespective of the exact signaling pathways involved in alum-mediated activation (12, 14, 15). In addition to alum, monophosphoryl lipid A (MPLA) is also used as an adjuvant in human vaccines (16). It is a derivate of lipid A from LPS that lacks toxicity but remains immunogenic (17, 18). The adjuvant is an agonist of TLR4, activating APCs through a TRIF-dependent signaling pathway (19). APC activation induced by MPLA results in increased production of TNF-a, IL-10, and IL-12 by APCs (20). MPLA as an adjuvant has been demonstrated to activate both the humoral and cellular arms of the immune response (21, 22).

As Freund's adjuvant is not licensed to be used in human vaccines, this study tested whether recombinant Sm29 antigen with MPLA or alum as an adjuvant could be effective in an animal model of *S. mansoni* reinfection. In addition, the immunological profiles induced by the vaccines were assessed. Our data show that immunization with Sm29 and alum was indeed able to induce partial protection against reinfection, reducing parasite burden by 29–37% in immunized mice. The Sm29 with MPLA formulation was unable to induce a reduction in worm burden, providing a powerful tool to understand why the Sm29 with alum formulation was effective. Further study comparing the two formulations suggested antibody production was important to the protective effect and higher antibody levels were observed in Sm29 and alum immunized animals. Although further investigation concerning the relationship between protection and antibody production is required, our data indicate that a vaccination formulation containing Sm29 and alum could potentially be used to control human schistosomiasis.

## Materials and methods

### Mice and parasites

Female BALB/c mice aged 6–8 weeks were obtained from the Centro de Pesquisas René Rachou (CPqRR)-FIOCRUZ (Fundação Oswaldo Cruz) animal facility, Brazil. LE strain *S. mansoni* cercariae were obtained by exposing infected *Biomphalaria glabrata* snails to light for 1–2 h to induce shedding. This *S. mansoni* strain is routinely maintained at the Lobato Paraense, CPqRR. The Ethics Committee of Animal Use (CEUA) of FIOCRUZ approved the protocols involving animals in this study under license number LW12/12.

### Antigen preparation

Recombinant Sm29 (rSm29) was produced and purified as previously described (4). Briefly, the protein was expressed in *Escherichia coli* BL21 bacteria carrying a pET21 expression vector containing the Sm29 cDNA. Expression was induced using 1 mM IPTG. After bacterial lysis, rSm29 was purified by affinity chromatography on nickel columns. The protein was then dialyzed against phosphate buffered saline (PBS) pH 7.2 and its concentration determined using a BCA Protein Assay Kit (Thermo Fisher Scientific, Waltham, MA, United States).

### Vaccination protocol

Before immunization, mice were sensitized using a prior *S. mansoni* infection with approximately 30 LE-strain cercariae. Forty-five days after infection, mice were treated with two doses of 800 mg/Kg Praziquantel as previously described (8). Fifteen days after treatment, mice were immunized (10 animals/group) with rSm29 (25 μg/animal) plus alum adjuvant -Alhydrogel (InvivoGen, San Diego, CA, United States) (1 mg/mouse/dose) or MPLA-SM VacciGrade (InvivoGen, San Diego, CA, United States) adjuvant (10 μg/mouse/dose). The control groups were inoculated with saline and either alum or MPLA-SM VacciGrade, respectively. Mice received three doses of each vaccine over the 15-d interval regimen, applied subcutaneously in the nape of the neck.

### Challenge infection and worm burden recovery

Animals were challenged by percutaneous infection with 100 cercariae (LE strain) 30 days after the final vaccine booster. Mice abdominal skin was shaved and exposed for 1 h to the cercariae. Fifty days after infection, adult worms were recovered from the portal veins as described by Pellegrino and Siqueira (23). The levels of protection were calculated as previously described (8). Fragments from the liver and intestine from control and experimental groups were collected after perfusion. These organs were weighted, digested in a solution of 10% KOH. The eggs were obtained and it number determined as previously described (8).

### Antibody assessment

The sera from the mice of each vaccinated group were obtained 15 days after each immunization dose. Enzyme-linked immunosorbent assays (ELISAs) were performed using the sera to evaluate the production of IgG, IgG1, IgG2a, and IgE antibodies specific to rSm29. Briefly, MaxiSorp 96-well microtiter plates (Nunc, Rochester, NY, United States) were coated with rSm29 at a concentration of 5 μg/mL (for IgG, IgG1, and IgG2a) or 1 μg/mL (for IgE) in carbonate-bicarbonate buffer (pH 9.6). These were left overnight at 4°C and were then blocked with 300 μL/well of phosphate-buffered saline (pH 7.2) with 0.05% Tween-20 (PBST) and 3% fetal bovine serum (FBS; GIBCO, Gaithersburg, MD, United States), for 2 h at 25°C (for IgG, IgG1, and IgG2a), or PBST plus 3% skim milk overnight at 4°C (for IgE). One hundred microliters of each serum sample was diluted 1:1,000 (for IgG, IgG1, and IgG2a) or 1:40 (for IgE) and added to the plates for 1 h. Finally, the plates were incubated with peroxidase-conjugated anti-mouse IgG (1:10,000), IgG1, (1:10,000), or IgG2a (1:8,000) (Southern Biotech, Birmingham, AL, United States), for 1 h at 25°C. For the IgE measurements, an anti-mouse biotin IgE (BD Biosciences, Franklin Lakes, NJ, United States) diluted to 1:250 was used. After incubation for 1 h at 25°C., an avidin conjugated to peroxidase (1:250) was added for 30 min. For all ELISAs, a TMB incubation was used to visualize antibody concentrations and was stopped with 5% sulfuric acid. Finally, the plates were read at 450 nm using an ELISA plate reader (Bio-Rad, Hercules, CA, United States). Endpoint antibody titers were determined using pool of sera samples from each group and a serial dilution range from 1:20 to 1:1,310,720.

### Cellular immune response and immunophenotypic analysis

To assess cytokine production, the spleens from at least five mice per group were collected 7 days after the final immunization dose. Red blood cells were lysed using ACK lysing buffer and the remaining splenic cells were washed twice with apyrogenic saline before being adjusted to 1 × 10^6^ cells/well. Cells were cultured in 5% CO_2_ at 37°C without stimulation (negative control), stimulated with anti-CD3 (1 μg/mL) as a positive control, or rSm29 (25 μg/mL). Culture supernatants were collected 24 or 72 h post stimulation to access the levels of IL-2 (24 h), IL-4 (24 h), IL-6 (24 h), IL-10 (72 h), IL-17 (72 h), IFN-γ (72 h), and TNF-α (24 h). Cytokine measurements were performed using an anti-mouse Th1/Th2/Th17 Cytometric Bead Array (CBA) Kit (BD Biosciences, Franklin Lakes, NJ, United States) following the manufacturer's protocol. The beads were quantified using a FACScalibur flow cytometer (BD Biosciences, Franklin Lakes, NJ, United States) and the data analyzed using FCAP Array Software (BD Biosciences, Franklin Lakes, NJ, United States).

The proportions of CD4^+^ lymphocytes producing IL-4, IFN-γ, or IL-10 were also evaluated via intracytoplasmic staining. Cells were initially processed as previously described, but were adjusted to 0.5 × 10^6^ cells per well and cultured for 18 h in the absence of polyclonal or antigen specific stimulation. To prevent cytokine secretion in the last 4 h, brefeldin A (1 μg/mL) was added to the culture. The cells were then blocked with either anti-CD16 or CD32 mAbs (clone 2.4G2; BD Biosciences, Franklin Lakes, NJ, United States). Surface molecules were stained by incubating the cells for 15 min with anti-CD4 conjugated to FITC (clone GK1.5; BD Biosciences, Franklin Lakes, NJ, United States) and anti-CD3 conjugated to biotin (clone 500A2; BD Biosciences, Franklin Lakes, NJ, United States). The cells were then washed with PBS (0.15 M) containing BSA (0.5%) and NaN_3_ (2 mM). Streptavidin PeCF594 (BD Biosciences, Franklin Lakes, NJ, United States) was then added. The cells were left for an additional 15 min at 4°C, washed with PBS and then fixed and permeabilized using Cytofix/Cytoperm fixation/permeabilization solution (BD Biosciences, Franklin Lakes, NJ, United States) for 20 min at 4°C. The cells were next incubated for 30 min with specific to anti-IL-4 conjugated to PE (clone 11B11; BD Biosciences, Franklin Lakes, NJ, United States), anti-IFN-γ conjugated to eFluor450 (clone XMG1.2; eBioscience, San Diego, CA, United States), and anti-IL-10 conjugated to APC (clone JES5-16E3; BD Biosciences, Franklin Lakes, NJ, United States) monoclonal antibodies. They were then washed with the same Cytofix/Cytoperm buffer (BD Biosciences, Franklin Lakes, NJ, United States) and sorted using an LSRFortessa BD flow cytometer (BD Biosciences, Franklin Lakes, NJ, United States). For the *ex vivo* analyses, splenocytes were incubated with CD16/CD32 and then anti-CD3 (clone 500A2; BD Biosciences, Franklin Lakes, NJ, USA), anti-CD4 (clone GK1.5; BD Biosciences, Franklin Lakes, NJ, United States), anti-CD11c (clone N418; eBioscience, San Diego, CA, United States), or anti-F4/80 conjugated to FITC (clone BM8; eBioscience, San Diego, CA, United States); anti-CD86 conjugated to PE (Accurate Chemical and Scientific Corporation, Westbury, NY, United States); anti-CD3 (clone 500A2; BD Biosciences, Franklin Lakes, NJ, United States), anti-CD27 (clone LG.7F9; Bioscience, San Diego, CA, United States), anti-F4/80 (clone BM8; Bioscience, San Diego, CA, United States), or anti-CD40 conjugated to biotin (eBioscience, clone 1C10); anti-CD62L conjugated to APC-Cy7 (clone MEL-14; BD Biosciences, Franklin Lakes, NJ, United States); anti-CD19 (clone 1D3; BD Biosciences, Franklin Lakes, NJ, United States), anti-CD127 (clone A7R34; BD Biosciences, Franklin Lakes, NJ, United States), or anti-CD11b conjugated to PE-Cy7 (clone M1/70; BD Biosciences, Franklin Lakes, NJ, United States); anti-I-A/I-E conjugated to Alexa 647 (clone M5/114.15.2; BD Biosciences, Franklin Lakes, NJ, United States); and anti-CD44 conjugated to Alexa 700 (clone IM7; eBioscience, San Diego, CA, United States) monoclonal antibodies. Then, cells were washed and incubated for 20 min with streptavidin APC-Cy7 or PeCF594. Finally, cells were fixed with 2% formaldehyde solution and acquired using an LSRFortessa (BD Biosciences, Franklin Lakes, NJ, United States). The data were analyzed using FlowJo software 7.6.3 (FlowJo LLC, Ashland, OR, United States). During analysis, sample acquisition that presented reduced number of acquired events, interruption of flow during acquisition or excessive number of doublets were excluded from the analysis. The staining panel used for each cell characterization is described in Supplementary Table 1.

### Statistical analysis

Data normality was tested using D'Agostino-Pearson omnibus tests. Based on a Grubbs' test, outliers were identified and eliminated. Statistical analyses were performed using ANOVA and Tukey‘s multiple comparison for the cytokine measurements and *ex vivo* analyses, or Student's *t*-tests for the parasitological analyses and antibody measurements. All calculations were performed using GraphPad Prism 7.02 (Graph-Pad Software, La Jolla, CA, United States).

## Results

### rSm29 formulated with alum induces partial protection against *S. mansoni* infection in pre-sensitized mice

Animals immunized with the vaccine formulation containing alum as an adjuvant (Sm29Alum) showed a protection level between 29 and 37%. A significant reduction in worm burden (25 ± 8; 26 ± 13 and 27 ± 10-trials 1, 2 and 3, respectively) after *S. mansoni* challenge was observed in these animals in comparison to PBS alum group (35± 9; 41 ± 8 and 38 ± 6-trials 1, 2, and 3, respectively). In contrast, immunization with the MPLA adjuvant (Sm29MPLA) did not confer any protection against reinfection challenge (Table 1). Sm29MPLA group presented 26 ± 11; 34 ± 15 and 33 ± 10 mean worm burdens and PBS/MPLA group presented 27 ± 12; 38 ± 16 and 31 ± 8 mean worm burdens after the first, second and third trials, respectively. In addition to the determination of the number of worms recovered, the number of parasite eggs trapped in the liver or crossing the intestine wall was determined. Agreeing with the significative reduction of the parasite burden, significantly fewer parasite eggs were observed in the liver of animals immunized with Sm29alum. But, any significant differences in the number of eggs per gram of intestine were observed between experimental and control groups, regardless the vaccine formulation.

**Table 1:** Worm recovery and protection level in Sm29 immunized groups.

	**Worm burden recovery**				
	**Male Mean ±SD**	**Female Mean ±SD**	**Total Mean ±SD**	**% of protection^b^**	**Egg/gram of liver Mean ±SD**	**% of reduction^b^**	**Egg/gram of intestine Mean ±SD**	**% of reduction^b^**
**TRIAL 1**^a^								
**Challenge control**							
ALUM	17 ± 5	18 ± 4	35 ± 9		ND		ND
Sm29 ALUM	12 ± 4	12 ± 4	25 ± 8	29% (*p* = 0.03)	ND		ND
MPLA	13 ± 6	15 ± 7	27 ± 12		ND		ND
Sm29 MPLA	13 ± 5	13 ± 6	26 ± 11	4% (NS)	ND		ND
**TRIAL 2**^a^								
**Challenge control**							
ALUM	21 ± 4	20 ± 4	41 ± 8		14241 ± 2695		8900 ± 1894
Sm29 ALUM	15 ± 7	15 ± 9	26 ± 13	37% (*p* = 0.02)	11004 ± 2776	23% (*p* = 0.04)	9727 ± *5248*	(NS)
MPLA	18 ± 8	20 ± 9	38 ± 16		10288 ± 2077		10400 ± 8712
Sm29 MPLA	18 ± 7	17 ± 8	34 ± 15	11% (NS)	13665 + 3391		9860 ± 4813	5 % (NS)
**TRIAL 3**^a^								
**Challenge control**							
ALUM	20 ± 5	17 ± 4	38 ± 6		43285 ± 21394		8265 ± 1536
Sm29 ALUM	15 ± 5	13 ± 5	27 ± 10	29% (*p* = 0.04)	22554 ± 5726	48% (*p* = 0.04)	7594 ± 4287	8%(NS)
MPLA	16 ± 5	15 ± 3	31 ± 8		58346 ± 28976		7568 ± 4768
Sm29 MPLA	14 + 6	15 + 7	33 + 10	(NS)	44069 ± 24010	24% (NS)	12042 + 5743	(NS)

a *For each vaccinated group: n = 10 mice*.

b *Comparison between total burden recovered from Sm29 ALUM or MPLA group and ALUM or MPLA control group*.

*NS, not significant*.

*ND, not determined*.

### Alum and MPLA adjuvants induce similar APC activation profiles

**Figure 1:**
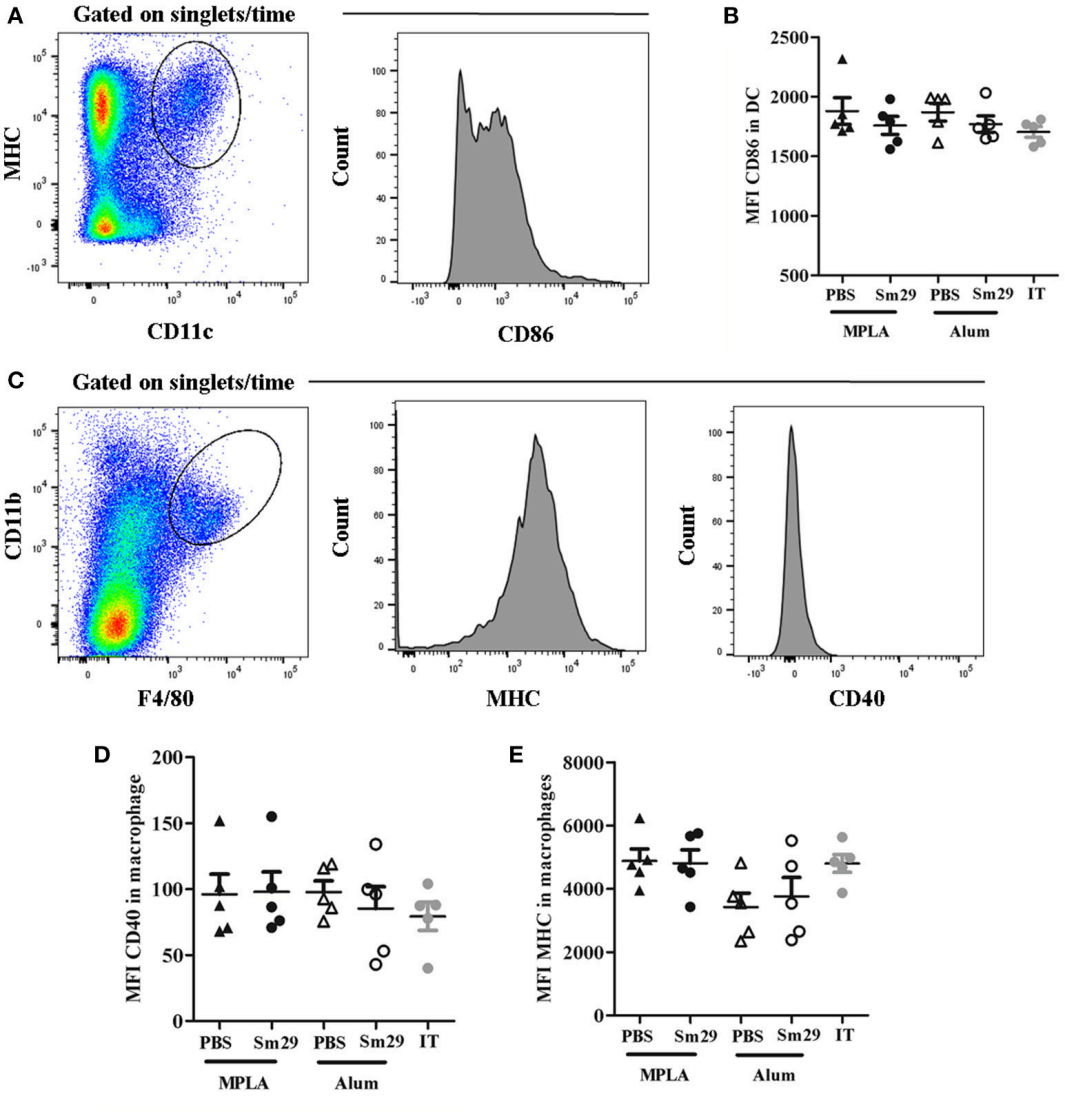
Activation status of dendritic cells (DC) and macrophages. Spleen from 5 to 6 animals was obtained for each group. Dendritic cells and macrophages were labeled to evaluate the expression of activation markers. For dendritic cells analysis, CD11c^+^ and MHC^+^ double positive cells were selected and the mean fluorescence intensity (MFI) of CD86 was determinate **(A)**. For macrophages, F4/80 and CD11b double positive cells were selected and the mean fluorescence intensity of MHCII and CD40 were evaluated **(C)**. Data of CD86 MFI in dendritic cells **(B)**, MHCII MFI **(D)** and CD40 MFI **(E)** in macrophages from animals of MPLA (closed triangles), Sm29 MPLA (closed circles), Alum (open triangles), Sm29 Alum (open circles) and infected/treated (gray circles) groups are represented on graphics. Results are representative of one from two independent experiments.

To evaluate how the adjuvants impact APC activation, we analyzed CD86 expression by DCs, and MHC II and CD40 expression by macrophages, isolated from the spleens of immunized, adjuvant control and infected and treated (IT) animals 7 d after the last immunization dose. No differences were found in the expression of CD86 on DCs, or in MHC II and CD40 expression on macrophages, between the groups (Figure 1). Although Alum Sm29 trigger a protective immune response, nor alum, neither MPLA presented an additional effect in activating DC and macrophages in mice that have been sensitized by a previous *S. mansoni* infection, suggesting that alum-induced protective mechanism is acting further upstream of the immune response.

### Both Sm29Alum and Sm29MPLA vaccine formulations induce a mixed Th1/Th2/Th17 type immune response

**Figure 2:**
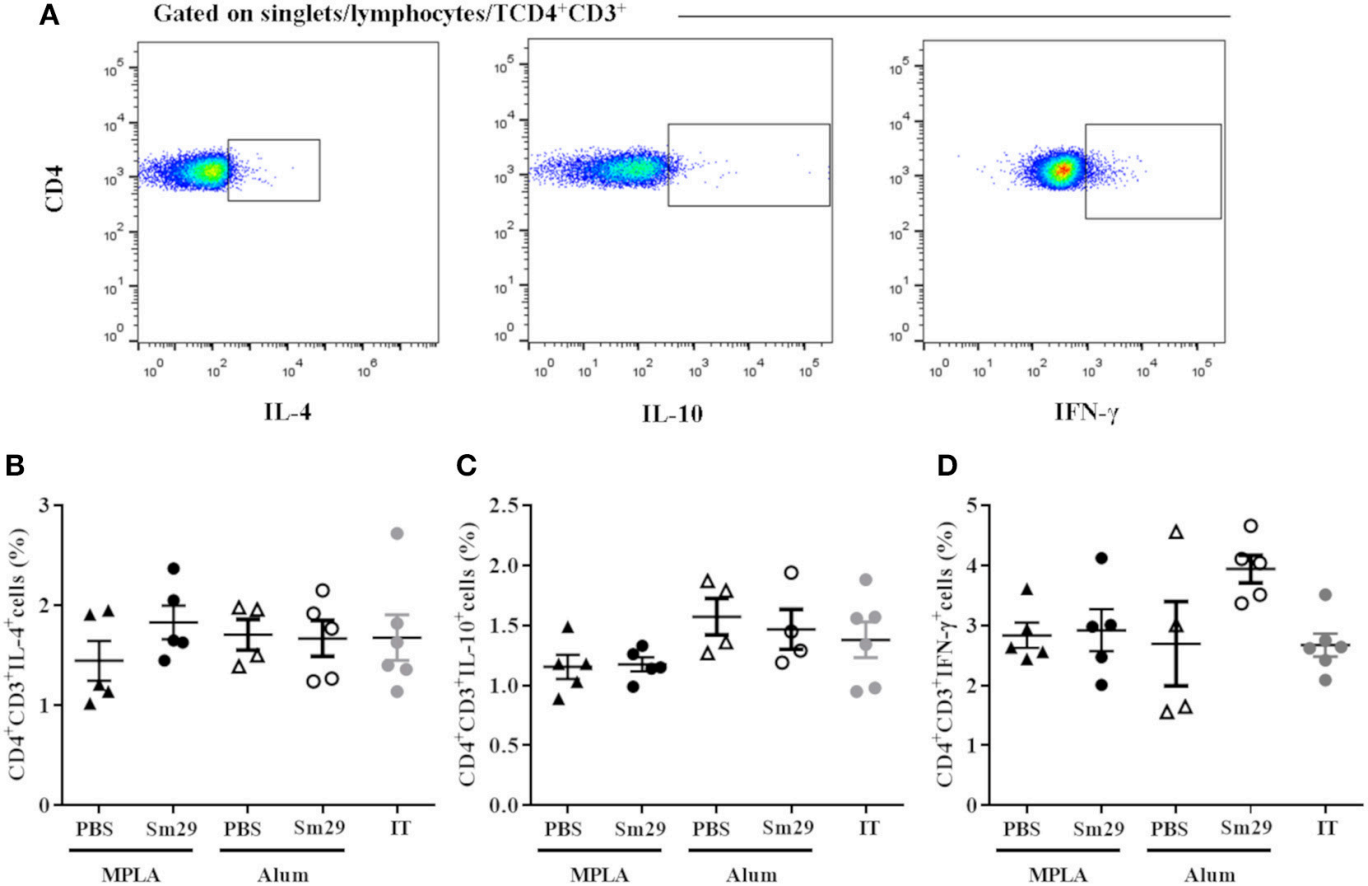
Profile of TCD4 subsets in animals immunized with Sm29 Alum and Sm29 MPL immunization. The percentage of CD4+IL-4+, CD4+IFN-γ+ and CD4+IL-10+ cytokines was evaluated by TCD4 intracellular staining in spleen cells from 5 to 6 animals of each group. Intracellular cytokine staining was performed in the absence of polyclonal or antigen specific stimulated to assesses differentiation induced by the vaccine formulation *in vivo*. Data analysis was carried out as demonstrated in **(A)** within singlet cells/lymphocyte region, cells expressing CD4 and CD3 molecules were selected and the percentage of CD4^+^ cells producing IL-4 **(B)**, IL-10 **(C)** and IFN-γ **(D)** was evaluated in MPLA (closed triangles), Sm29 MPLA (closed circles), Alum (open triangles), Sm29 Alum (open circles) and infected/treated (gray circles) groups. Results are representative of one from two independent experiments. No significant differences between groups were observed.

**Figure 3:**
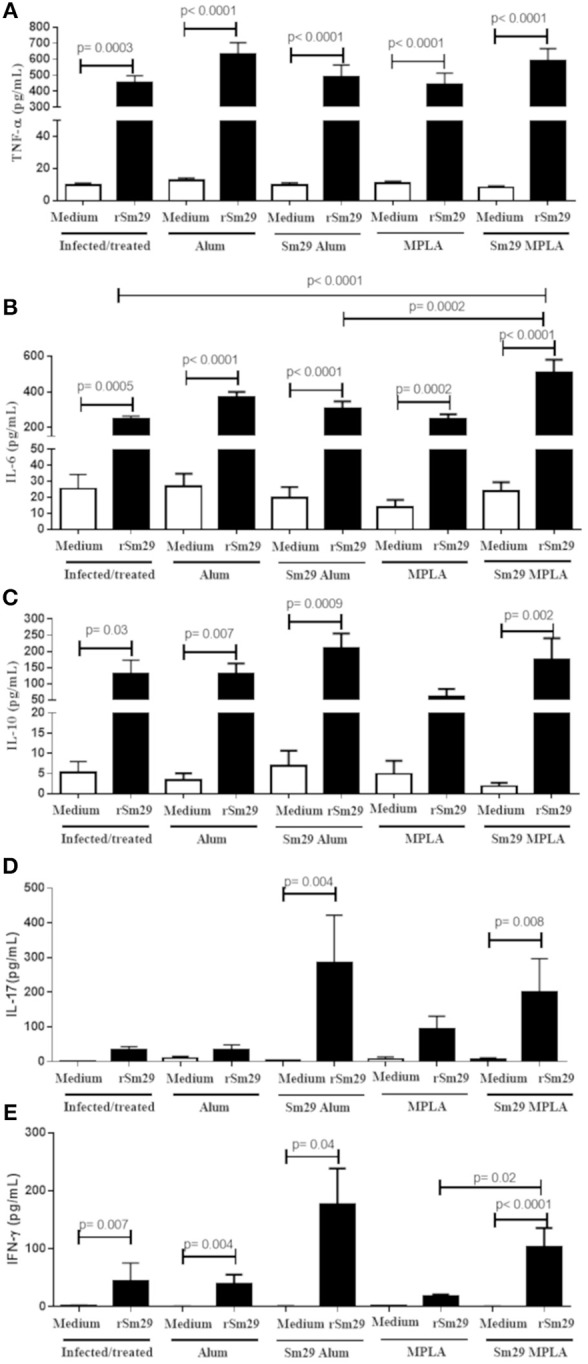
Cytokine production by spleen cells culture of immunized mice. Spleen from animals of MPLA (*n* = 11); Sm29 MPLA (*n* = 14); Alum (*n* = 11), Sm29 Alum (*n* = 15) and Infected and treated (*n* = 11) groups were obtained. Spleen cells cultured with (black bars) or without (open bars) rSm29 (25 μg/mL) stimulation was assessed to determine cytokine production. The levels of TNF-a **(A)**, IL-6 **(B)**, IL-10 **(C)**, IL-17 **(D)**, and IFN-γ **(E)** were measured by the CBA Th1/Th2/Th17 Kit. Statistically significant differences are denoted on the graphics. Bars represents the mean + SD values of three independent experiments.

Next, the cellular immune responses triggered by Sm29Alum, Sm29MPLA immunization and their respective adjuvant controls were assessed to understand the cytokine profiles involved in the protective (Sm29Alum) and non-protective vaccine formulation (Sm29MPLA). The impact of APC activation in T cell differentiation was also evaluated by intracellular staining using flow cytometry. We found that similar proportions of CD4^+^ cells producing IL-4, IFN-γ, and IL-10 were observed in the spleens of mice immunized with Sm29MPLA or Sm29Alum and their adjuvant control groups (Figure 2). Also, no differences in the proportion of CD4+ cells producing cytokine was observed in infected and treated group in comparison to other groups. Regarding cytokine production by spleen cells, Sm29 *in vitro* stimulation leaded to an increased production of TNF-α and IL-6 in all groups. IL-10 and IFN-γ production was also increased in all groups, except MPLA (Figure 3). Sm29MPLA immunization significantly increased IFN-γ production in comparison to MPLA inoculated animals (Figure 3E). Significant IL-17 production was only observed in the groups of animals that received Sm29 immunization. IL-6 levels were significantly higher in Sm29MPLA group in comparison to Sm29Alum and infected and treated groups (Figures 3B,D). Cytokine production under antigen specific recall was expected in the adjuvant control groups and in infected and treated mice (IT), since those animals were sensitized by a previous infection. However, as rSm29 was produced in bacteria, endotoxin contamination might also have contributed to increase the production of LPS-induced cytokines in stimulated cells. IL-4 and IL-2 levels were below CBA detection levels and could not be determined. Together, these results indicate that Sm29 immunization in association with Alum or MPLA does not significantly alter the immune profile induced by a previous infection with *S. mansoni*, and induces a Th1/Th2/Th17 mixed type of immune response.

### Mice immunization with Sm29 decreases the percentage of B cells in spleen, but fails to increase the percentage of memory cells

**Figure 4:**
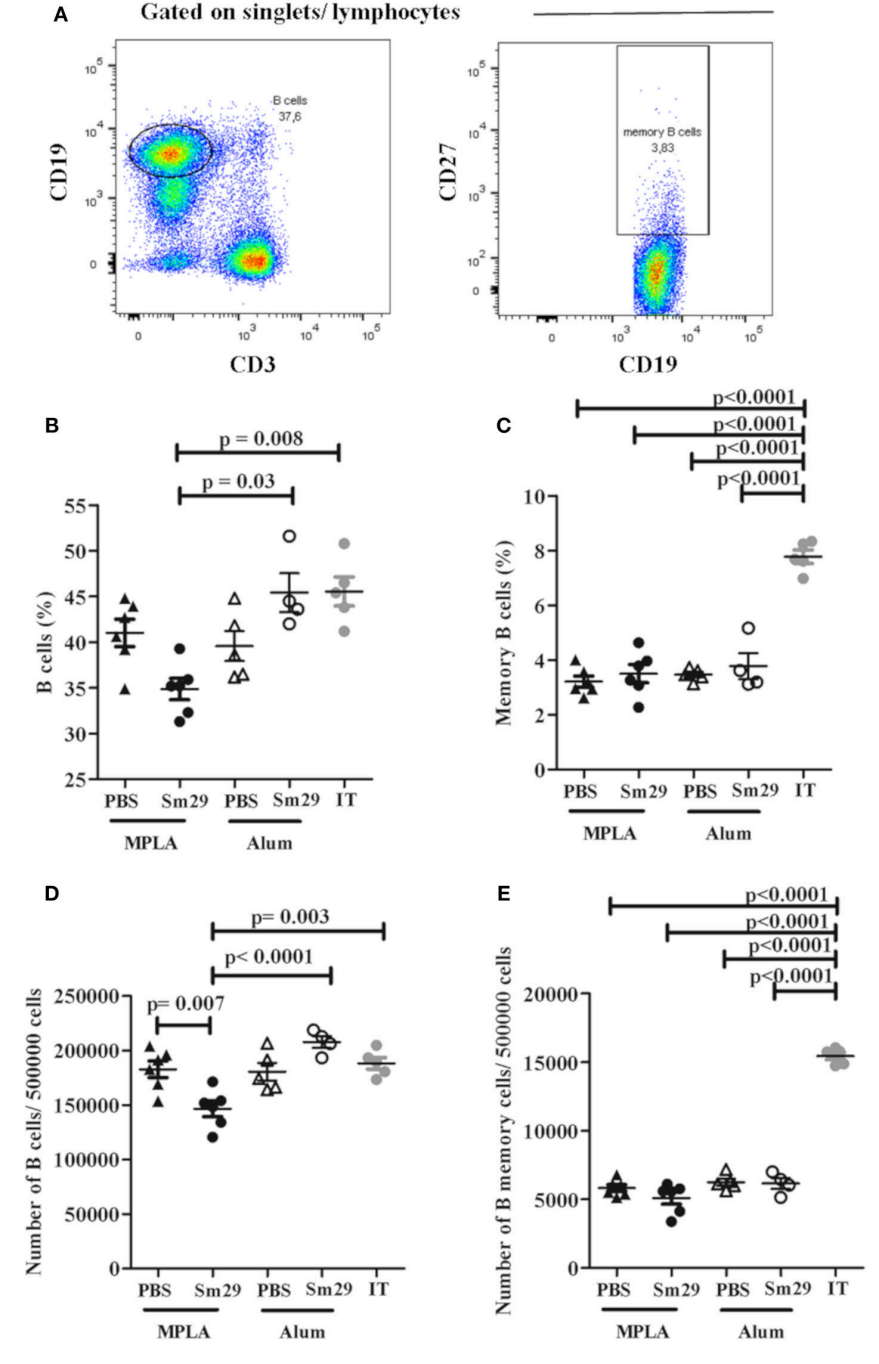
Frequency of B memory cells in immunized mice. Spleen from 5 to 6 animals was obtained for each group to determine the frequency of B memory cells. Data analysis was carried out as demonstrated **(A)**: within singlet cells/lymphocyte population, CD19^+^ and CD3^−^ cells were selected and the percentage of B cells was determined. B memory cells were assessed by determining CD19^+^CD27^+^cell frequency. Data represents percentage and number of B Cells **(B,D)** and B memory cells **(C,E)** in animals from MPLA (closed triangles), Sm29 MPLA (closed circles), Alum (open triangles), Sm29 Alum (open circles) and infected/treated (gray circles) groups. Statistically significant differences are denoted on the graphics. Results are representative of one from two independent experiments.

**Figure 5:**
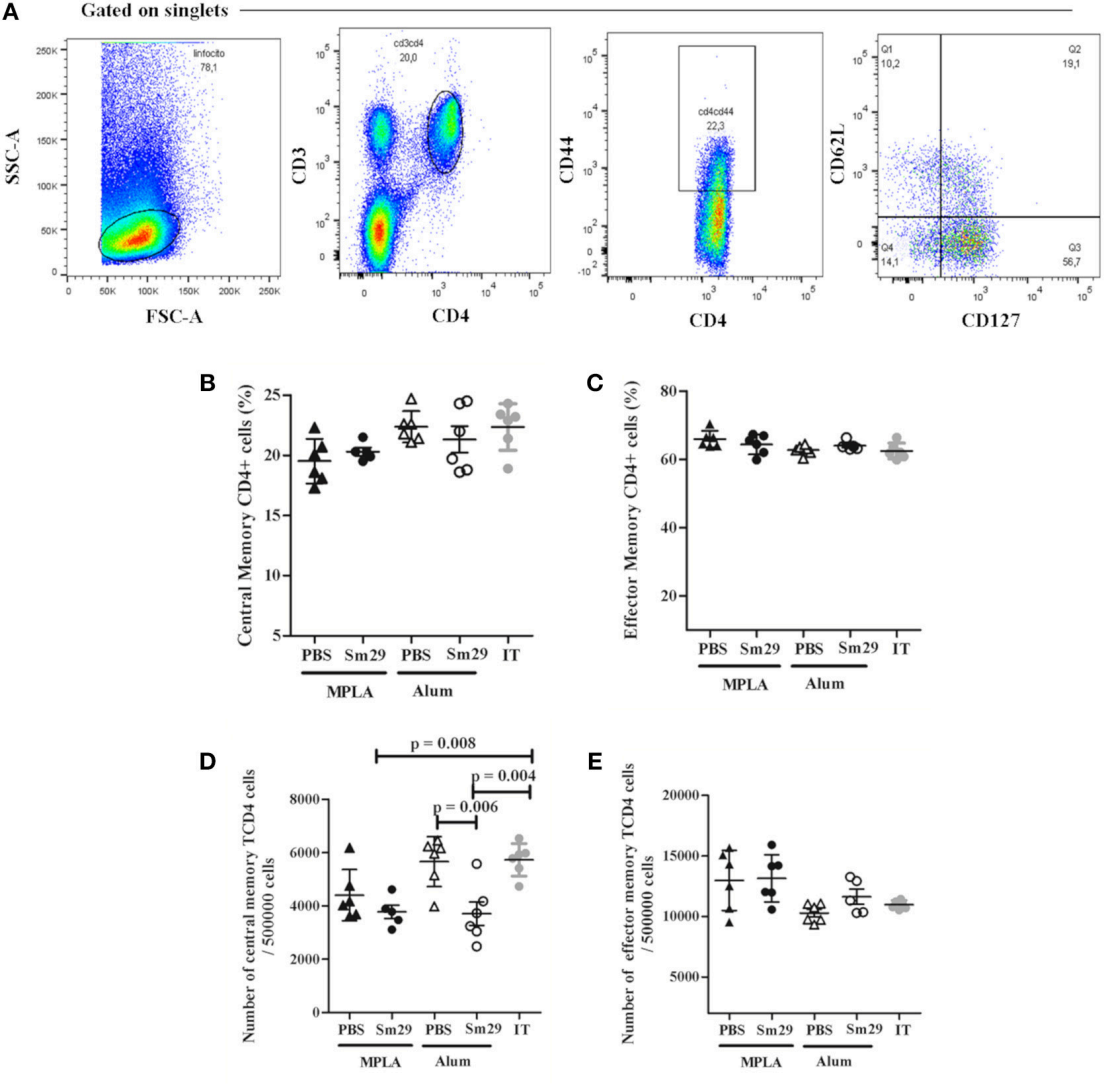
Frequency of T memory cells in immunized mice. Spleen from 5 to 6 animals was obtained for each group to determine the frequency of T memory cells. Data analysis was carried out as demonstrated **(A)**: within singlet cells/lymphocyte population, CD4^+^CD44^high^ cells were selected and, within that population, the percentage of CD127^+^CD62^low^ cells representing CD4^+^ T effector memory cells, CD127^+^CD62^high^ population representing the CD4^+^ T central memory cells were determined. Data represents percentage and number of CD4^+^ T central memory cells **(B,D)** effector memory cells **(C,E)** in animals from MPLA (closed triangles), Sm29 MPLA (closed circles), Alum (open triangles), Sm29 Alum (open circles) and infected/treated (gray circles) groups. Results are representative of one from two independent experiments.

The percentage of B and T memory cells was determined as demonstrated in Figures 4A, 5A. A decrease in the percentage and absolute number of B cells in spleen was observed in mice immunized with Sm29 MPLA in comparison to Sm29 Alum and IT groups (Figures 4B,D). Significant decrease in the proportions and number of memory B cells, characterized as CD3^−^CD19^+^CD27^+^ cells was observed in all groups in comparison to IT group (Figures 4C,E). Nevertheless, any difference in the proportions of CD4^+^ central memory T cells (CD3^+^CD4^+^CD44^hi^CD127^+^CD62L^+^) or effector memory T cells (CD3^+^CD4^+^CD44^hi^CD127^+^CD62L^−^) was observed between groups (Figures 5B,C). Although a significant reduction in the number of CD4^+^ central memory cells was observed in Sm29 immunized mice in comparison to IT group (Figure 5D). No significant reduction in the number of CD4^+^ effector memory cells was observed (Figure 5E).

### The Sm29Alum vaccine formulation promotes higher titers of anti-Sm29 antibodies

**Figure 6:**
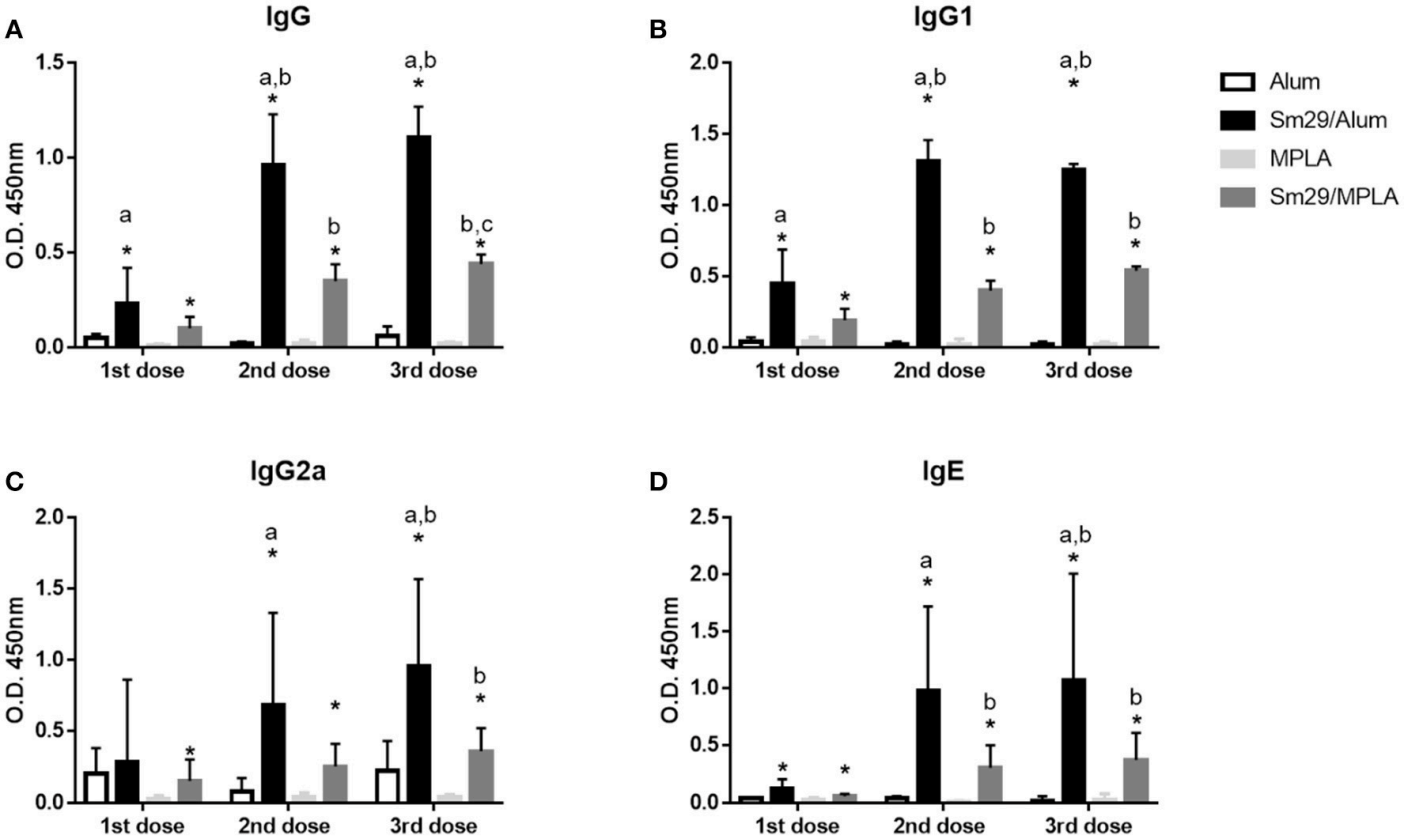
Production of Sm29-specific antibodies in immunized mice. Sera from mice were obtained 15 days after each immunization dose and were assessed to determine the levels of IgG **(A)**, IgG1 **(B)**, IgG2a **(C)**, and IgE **(D)** antibodies against rSm29 in the animals inoculated with alum (open bars), MPLA (bright-gray bars), Sm29Alum (black bars) or Sm29/MPLA (dark-gray bar). Significant differences between adjuvant control and experimental groups are indicated by an asterisk (*p* < 0.05). Significant difference between Sm29/Alum and Sm29/MPLA groups are denoted by the letter a (*p* < 0.05). Significant differences compared to the first and are second immunization dose are denoted by letters b and c, respectively. Results are representative of one from two independent experiments.

To more fully establish the role of antibody production in protection against *S. mansoni* reinfection, we examined the humoral response in more detail for Sm29Alum and Sm29MPLA. Both formulations induced increased production of IgG, IgG1, IgG2a, and IgE antibodies specific for rSm29 relative to adjuvant control groups. In particular, mice immunized with the Sm29Alum formulation showed higher production of IgG, IgG1, and IgE after all immunization doses when compared to PBS/Alum group. However, increased IgG2a production was only observed after the second immunization dose. The Sm29MPLA formulation also promoted increased production of anti-Sm29 IgG, IgG1, IgG2a, and IgE antibodies after each of the three vaccine doses relative to the PBS/MPLA group. Further vaccination boost increased the levels of Sm29-specific IgG and IgG1 antibodies in the Sm29Alum group and IgG, IgG1, and IgE antibodies in the Sm29MPLA group. No increases in antibody levels were observed after the second vaccine boost (third vaccine dose) in either group, except for IgG levels in Sm29MPLA immunized animals (Figure 6). Importantly, while both formulations were able to induce the production of anti-Sm29 antibodies, antibody levels were much higher in the Sm29Alum group compared to the Sm29MPLA group (Figure 6). As an indicative of immune response profile, we also evaluated the IgG1 and IgG2a ratio over the immunization protocol (Supplementary Figure 1). At the beginning of the immunization protocol, the IgG1/IgG2a ratio suggests a Th2 type of immune response in animals immunized with Sm29 alum formulation, however following vaccine boosts, a decrease IgG1/IgG2a ratio is observed in these animals and no significant differences between Sm29 Alum and Sm29MPLA groups are observed. These data corroborate the observation of a mixed Th1/Th2/Th17 immune profile observed after the third immunization dose in response to both vaccine formulations. Finally, we also assessed overall endpoint antibody titers between the groups (Supplementary Figure 2). Animals immunized with Sm29Alum had IgG, IgG1, IgG2a, and IgE endpoint titers of 81,920; 327,680; 5,120, and 640, respectively. This endpoint antibody titers were at least twice as high as the endpoint titers observed in Sm29MPLA immunized mice which was 20,480; 81,920; 2,560 and 320 for IgG, IgG1, IgG2a, and IgE, respectively. Due to a previous infection with *S. mansoni*, animals from PBS/alum or PBS/MPLA presented antibodies specific for the recombinant Sm29 in their sera. PBS/Alum group presented 2,560(IgG), 2,560 (IgG1), 5,120 (IgG2a), and 20 (IgE) Sm29-specific endpoint antibody titers. Sm29-specific endpoint titers in PBS/MPLA were 320 (IgG), 2560 (IgG1), 320 (IgG2a), and 40 (IgE). Altogether, these results suggest that the effectiveness of the Sm29Alum vaccine formulation may be due to increased antibody production that lead to more Sm29-specific Ig.

## Discussion

The Sm29 tegument protein from *S. mansoni* (5) has been considered a promising candidate that could serve as a vaccine against schistosomiasis. This is primarily due to its ability to reduce parasite burden across a range of vaccine strategies and formulations (5, 24, 25). The ability of Sm29 to induce protection against reinfection has recently been described, reinforcing its potential as a vaccine candidate (8). Here, we have built on this initial study that used Freund's adjuvant to induce protective immunity by testing vaccines containing Sm29 with two adjuvants approved for use in humans, alum and MPLA. Our study has shown that the Sm29Alum formulation elicited partial protection, whereas Sm29MPLA did not. This has provided a valuable tool to investigate the immune responses associated with successful vaccine protection against *S. mansoni*.

Adjuvants are important components of vaccine formulation and are responsible for inducing local inflammation that favors APC activation and differentiation (26). However, alum and MPLA belong to different classes of adjuvants, with MPLA acting as a TLR4 agonist (19). Alum is not recognized by any known innate sensor and instead leads to tissue damage and the release of uric acid that is detected by cells of the innate immune system (12). Despite using different mechanisms that can be either direct or indirect, these adjuvants activate innate cells, including professional antigen presenting cells, linking the innate response to adaptive response. This is essential for immunity induced by vaccines (10–13). Under our immunization protocol, both alum and MPLA showed no additional effect in activating DCs and macrophages, since the level of expression of activation markers in both cell types are similar to the ones observed in not immunized infected and treated mice. In addition to a role in activating APCs, the induction of a cytokine profile capable of eliciting protective effector function is also critical. Our cytokine measurements using the supernatants of cultured splenocytes isolated from animals of each groups, demonstrates a significant production of IL-6, IL-10 and TNF-a in response to rSm29 stimulation in all groups. Endotoxin contamination in the rSm29 might have contributed to increase the production of these cytokines *in vitro*. But it was previously demonstrated that Sm29 itself stimulate the production of LPS-induced cytokines, in TLR4KO mice (5). In addition, no differences were found in the proportions of IFN-γ, IL-4, or IL-10 producing CD4^+^ T cells. But Sm29 immunization regardless the adjuvant used induces a significant production of IL-17 under specific stimulation. These observations demonstrate that, under a context of prior sensitization with the parasite, both formulations induce a mixed Th1/Th2/Th17 immune response. Analysis of the IgG1/IgG2a ratio corroborates this observation. It has previously been shown that Sm29 immunization of naive animals induces a Th1-type immune response characterized by increased IFN-γ production (5, 24). However, it was recently demonstrated that there is also production of Th2 cytokine markers by spleen cells in response to rSm29 after challenge (25). These discrepancies may be due to the fact that we used BALB/c mice instead of C57BL/6 mice to test the protection levels induced by vaccination. BALB/c mice produce higher levels of IL-13 and IL-4 than C57BL/6 mice in response to parasite soluble eggs-antigens stimulation after *S. mansoni* infection and reinfection (27). Is also worth mention that although we didn‘t measured cytokine after reinfection, in our model mice were primed with a *S. mansoni* infection before immunization. Therefore, the experimental model used in our study appears more prone to a Th2-type response, even before immunization.

In our study we have observed a decreased number of B cell in spleen from mice immunized with Sm29 MPLA, while the Sm29 alum immunization didn‘t change the proportion and number of B cells in comparison with mice from the infected and treated group. However, immunization significantly reduced the number of B memory cells. Alum has been described as an adjuvant that activates Th follicular cells which is important to memory B cells and long-lived plasma cells differentiation (28). Once we had not observed increased percentage of memory B cells in Sm29 alum immunized animals, B cells in the spleen of immunized mice might be differentiating into plasma cells rather than into memory cells. Therefore, the protective effect mediated by Sm29Alum is likely to be linked to antibody production rather than memory function. Our data have also revealed the importance of antibody production in eliciting effective protection. Several studies have also demonstrated that antibodies play a key role in the protection induced by other schistosome vaccine candidates (29–31). In particular, our results demonstrate that the protective vaccine formulation (Sm29Alum) induced increased production of antibodies specific to Sm29, with endpoint titers at least twice as high as those observed in the non-protective formulation (Sm29MPLA). However, it is important to state that although Sm29 is one of the major antigen targets in the sera of mice, rats, and humans infected with *S. mansoni* (32), high production of anti-Sm29 antibodies does not necessarily correlate with resistance to infection. Despite this, isotype specific antibody responses have been associated with resistance in humans. Individuals naturally resistant to *S. mansoni* infection and reinfection produce high levels of Sm29-specifc IgG1, or IgG1 and IgG4, respectively (4, 33). We also observed higher endpoint titers of Sm29-specific IgG1 (that correlate with the human IgG4 isotypes) and IgE antibodies in the mice that received the protective Sm29Alum formulation. A previous study using Sm29 formulated with Freund's adjuvant in mice sensitized by prior infection also led to increased production of specific anti-Sm29 antibodies (8). In the study, three doses of vaccine were necessary to induce protection, with the levels of specific IgG1 and IgG2a increasing with each boost and the highest titers reached after the third dose (8). Together, these data suggest that antibodies are associated with the protective immunity shown by Sm29Alum against *S. mansoni* reinfection in mice. Despite its ability to induce a protective immune response in mice, Sm29 alum immunization also induces significant production of specific IgE. IgE against vaccine candidates is a safety concern when developing vaccine formulations. IgE against vaccine candidates might induce allergic reactions in vaccinees, as the systemic urticarial reaction observed in the Na-ASP2 hookworm vaccine clinical trial (34). Therefore, Sm29 alum allergenicity should be tested before moving for clinical trials.

No difference in the percentage of B memory cells was observed between the immunized groups. We also did not observe any differences in the proportions of CD4^+^ T memory cells between the Sm29Alum and Sm29MPLA groups. Previous human studies in *S. haematobium* endemic areas have demonstrated that an increase in the proportion of CD4^+^ T memory cells associates with age and resistance to reinfection (35). However, in our study, we did not observe any association between T or B memory cells and the protective response, but increased numbers of B and T memory cells are observed in infected and treated group in comparison to Sm29 immunized animals, indicating that memory cells reflect previous exposure to the parasite rather than protection.

The use of the mice model in the study of vaccine formulations against schistosomiasis was criticized in a review published recently (36), due to physiological characteristics of the mouse that might reduce parasite migration and maturation in a non-specific way. However, is worth mention that in our model, the control group (adjuvant group) received the same treatment as the experimental group, except for the absence of the antigen in the vaccine formulation. Therefore, the partial protection observed in Sm29Alum experimental group can only be attributed to the acquired immune response triggered by the vaccine. Mice also represent a valuable animal model to explore the immune mechanism associated with protection, in contrast to other animal models a huge amount of tools, as monoclonal antibodies, knockout and conditional knockout mice are available. The knowledge generated using mice models are useful to screen vaccine candidates and rationally design a vaccine formulation to be used in humans. However, once promising antigen have been identified and the mechanisms of protective immunity have been clarified, vaccine formulations should also be evaluated in other animal models before moving forward to clinical trials.

In conclusion, our study has shown that a vaccine formulation containing Sm29 and alum is able to confer protection against *S. mansoni* reinfection in mice. Our further functional investigation suggests that this may be due to an association between Sm29-specific antibodies and protective immunity, although further work to clarify this relationship is required. Nevertheless, our data indicate that a vaccination formulation containing Sm29 and alum could contribute to the control of human schistosomiasis and should be examined further.

## Author contributions

CA, NA, WB, and MM participated in the experiments. CA, SO, and CF performed data analysis. CA and CF designed the study.

### Conflict of interest statement

The authors declare that the research was conducted in the absence of any commercial or financial relationships that could be construed as a potential conflict of interest.

## Supplementary material

Supplementary Figure 1 IgG1/IgG2a ratio in Sm29 immunized mice. Sera from mice immunized with Sm29 alum (gray bar) or Sm29 MPLA (white bars) were obtained 15 days after each immunization dose and were assessed to determine the levels of IgG1 and IgG2a antibodies against rSm29. IgG1/IgG2a ratio was determined by dividing absorbance values obtained in IgG1 and IgG2a ELISA. Significant differences between groups and between immunization doses are indicated in the graphic. Two asterisks (^**^) indicate *p-value* < 0.01.

Supplementary Figure 2 Sm29-specific endpoint antibodies titers in immunized mice. Pool of sera from mice of Alum (triangle), MPLA (circle), Sm29 Alum (inverted triangle) and Sm29 MPLA (square) groups were prepared using sera obtained 15 days after the last immunization dose. Sera serial dilution begging in 1:20 (IgE) and 1:80 (IgG, IgG1 and IgG2a) were used to determine endpoint antibody titers. Threshold was calculated using the mean absorbance value of the blank wells plus two standard deviation and is indicated in the graphic by the dotted line.

Supplementary Table 1 Antibodies panel for cell phenotyping.
