# Supplementary figures and images for: A Strong Humoral Immune Response Induced by a Vaccine Formulation Containing rSm29 Adsorbed to Alum Is Associated With Protection Against Schistosoma mansoni Reinfection in Mice
Source: Front Immunol. 2018 Nov 2;9:2488. doi: 10.3389/fimmu.2018.02488 (PMC6224358; doi:10.3389/fimmu.2018.02488)

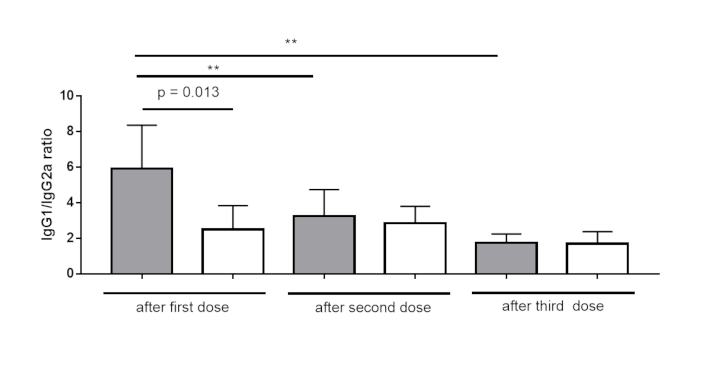

Supplement: Supplementary Figure 1 — IgG1/IgG2a ratio in Sm29 immunized mice. Sera from mice immunized with Sm29 alum (gray bar) or Sm29 MPLA (white bars) were obtained 15 days after each immunization dose and were assessed to determine the levels of IgG1 and IgG2a antibodies against rSm29. IgG1/IgG2a ratio was determined by dividing absorbance values obtained in IgG1 and IgG2a ELISA. Significant differences between groups and between immunization doses are indicated in the graphic. Two asterisks (**) indicate p-value < 0.01. [file Image_1.TIFF]

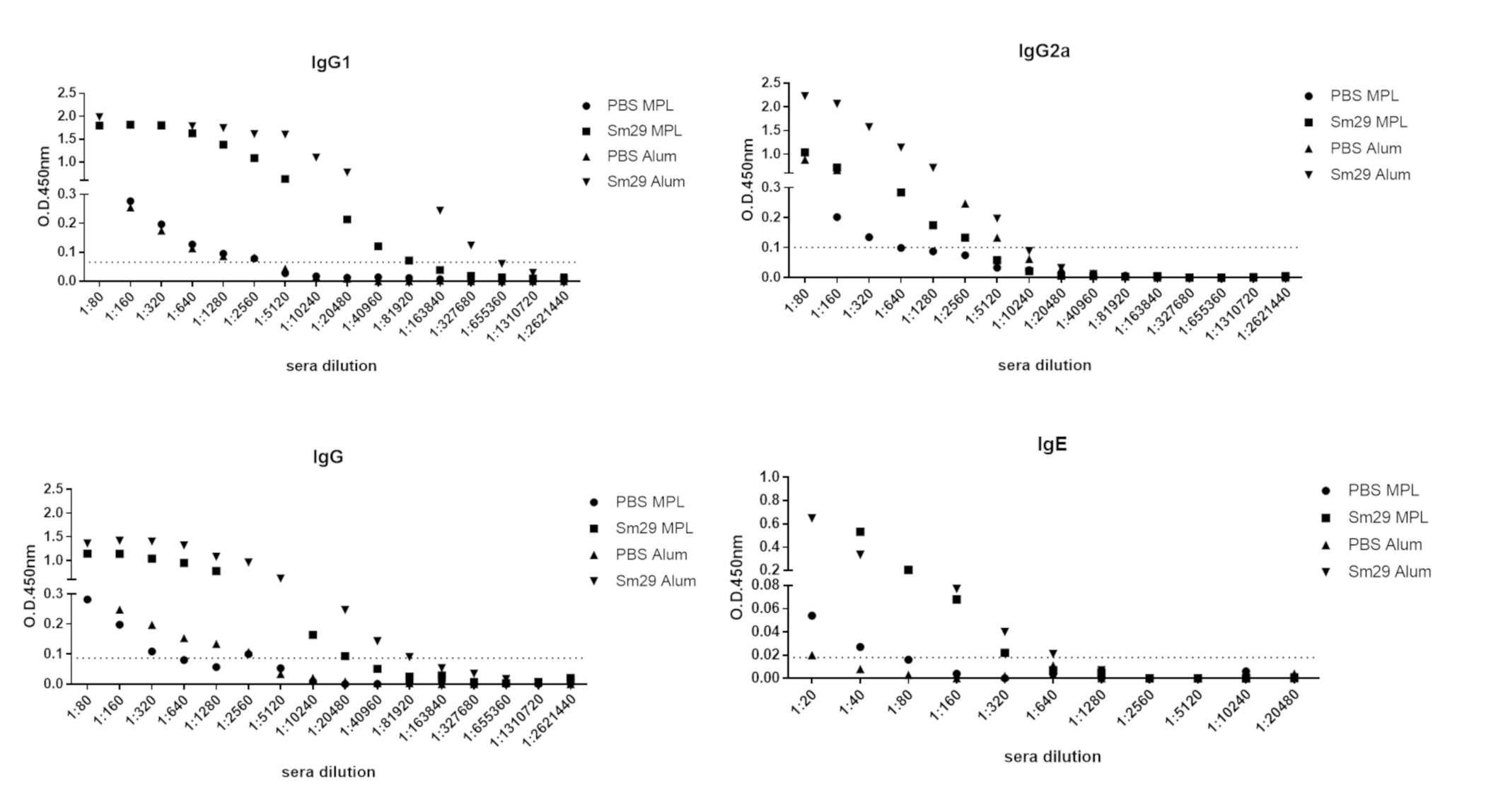

Supplement: Supplementary Figure 2 — Sm29-specific endpoint antibodies titers in immunized mice. Pool of sera from mice of Alum (triangle), MPLA (circle), Sm29 Alum (inverted triangle) and Sm29 MPLA (square) groups were prepared using sera obtained 15 days after the last immunization dose. Sera serial dilution begging in 1:20 (IgE) and 1:80 (IgG, IgG1 and IgG2a) were used to determine endpoint antibody titers. Threshold was calculated using the mean absorbance value of the blank wells plus two standard deviation and is indicated in the graphic by the dotted line. [file Image_2.TIF]
