# Supplementary material for: A Strong Humoral Immune Response Induced by a Vaccine Formulation Containing rSm29 Adsorbed to Alum Is Associated With Protection Against Schistosoma mansoni Reinfection in Mice
Source: Front Immunol. 2018 Nov 2;9:2488. doi: 10.3389/fimmu.2018.02488 (PMC6224358; doi:10.3389/fimmu.2018.02488)
Supplement: Supplementary Table 1 — Antibodies panel for cell phenotyping. [file Table_1.DOC]

| **Panel** | **Monoclonal antibody** | **Fluorochrome** | **Streptavidin** |
| --- | --- | --- | --- |
| **Intracytoplasmic cytokine staining** | Anti-CD4 | FITC |  |
|  | Anti-CD3 | Biotin | PeCF594 |
|  | Anti-IL-4 | PE |  |
|  | Anti-IFN-ɣ | eFluor450 |  |
|  | Anti-IL-10 | APC |  |
| **Memory T Cells** | Anti-CD4 | FITC |  |
|  | Anti-CD3 | Biotin | PeCF594 |
|  | Anti-CD44 | Alexa700 |  |
|  | Anti-CD62L | APC-Cy7 |  |
|  | Anti-CD127 | PE-Cy7 |  |
| **Memory B Cells** | Anti-CD3 | FITC |  |
|  | Anti-CD19 | PE-Cy7 |  |
|  | Anti-CD27 | Biotin | APC-Cy7 |
| **Macrophage** | Anti-F4/80 | FITC |  |
|  | Anti-CD11b | PE-Cy7 |  |
|  | Anti-I-A/I-E | Alexa647 |  |
|  | Anti-CD40 | Biotin | APC-Cy7 |
| **Dendritic Cells** | Anti-CD11c | FITC |  |
|  | Anti-I-A/I-E | Alexa647 |  |
|  | Anti-CD86 | PE |  |
|  |  |  |  |

**Table S1** – Antibodies panel for cell phenotyping.
